# Treatment Outcome and Adverse Events of Tenofovir Disoproxil Fumarate Based Regimens as Compared to Zidovudine Based Regimens Among People Living with HIV/AIDS: A Systematic Review and Meta-Analysis of Observational Studies

**DOI:** 10.2174/1874613601812010038

**Published:** 2018-05-31

**Authors:** Adane Teshome Kefale, Tegene Legese Dadi, Tessema Tsehay Biru, Teshale Ayele Mega

**Affiliations:** 1 College of Health Sciences, Mizan-Tepi University, Tepi, Ethiopia; 2 Institute of Health Sciences, Jimma University, Jimma, Ethiopia

**Keywords:** Tenofovir, TDF, Zidovudine, HIV/AIDS, Treatment outcome, ZDV group

## Abstract

**Background:**

Findings from different studies report inferior clinical and virologic efficacy with TDF/3TC/NVP. But, some studies show that, there was no statistically significant difference in mortality among ZDV and TDF based regimens. The objective of this review was to systematically identify, appraise and synthesize the best available evidence on efficacy and safety of TDF based regimen as compared to ZDV based regimens.

**Methods:**

A three-step search strategy was used to locate published and unpublished studies. First, an initial limited search of google was undertaken followed by analysis of text words. A second extensive search was undertaken. We searched the PubMed, EMBASE, Google Scholar, Medline, and CINHAL. We did the initial search for articles on July 11-18, 2016, and updated the results on May 13, 2017.Third, the reference lists of all identified articles was searched for additional studies.

**Results:**

ZDV based regimens had better outcome on prevention of mortality (OR=1.31, 95%CI (1.14, 1.50), I^2^ = 0%, Chi^2^ = 2.51), and lower virologic failure (OR = 1.44, 95% CI [1.18, 1.76], chi^2^ = 5.91, P= 0.003, I^2^ =83%) while, TDF based regimens were more tolerable (OR=0.15, 95%CI (0.08, 0.30), I^2^ = 40%, Chi^2^ = 3.31). The difference in incidence of opportunistic infection is not significant (OR = 0.83, 95% CI [0.52, 1.32], chi^2^ = 0.11, P= 0.42, I^2^ =0%).

**Conclusion:**

There is lower mortality and lower virologic failure in ZDV group, but better safety profile among TDF based regimens.

## 1 INTRODUCTION

Although introduction of potent combination Antiretroviral Therapy (cART) into clinical practice had advanced the treatment of Human immunodeficiency virus (HIV) infection [1, 2], the safety and efficacy of these agents was always a concern. The first decade after the advent of effective cART was marked by improving safety, efficacy, tolerability and ease of administration among regimens [3]. This resulted in rapidly emerging scientific understanding of HIV treatment, care and dynamic scale up efforts in resource limited settings with subsequent periodic updates of World Health Organization (WHO) guidelines [4].

Based on evidences of efficacy and safety, the 2010 WHO HIV treatment guideline recommended either tenofovir disoproxil fumarate (TDF) or zidovudine (ZDV) based combinations to be utilized as a first line agents in resource limited settings [5]. Similarly, the 2016 guideline had established TDF/3TC/EFV to be the preferred first line agent, with TDF/lamivudine(3TC)/nevirapine(NVP) or ZDV/3TC/efavirenz(EFV) or NVP as an alternative first line agents [6]. Consequently, many countries made a progress towards initiating first line cART with TDF backbone in HIV naïve patients, although 27% of HIV infected patients in sub-Saharan Africa still received ZDV based regimens [7].

These cART regimens saved hundreds of thousands of lives and provide hope to millions of others [8]. Despite achievements in scaling up access to cART, reduction in HIV related morbidity and mortality accompanied with significant increment in life expectancy of PLWHA [9-11], there were concerns regarding efficacy, safety and tolerability of these agents. Previous studies linked TDF based regimens with nephrotoxicity and reduction in bone mineral [12-14]. Finding from large Nigerian cohort showed inferior clinical and virologic efficacy of TDF when combined with NVP [15]. However, other studies reported that there was no statistically significant difference in all-cause mortality [16] and risk of HIV-1 disease progression or death among ZDV and TDF based regimens [17, 18]. In contrary, TDF based regimens were reported to have durable antiviral response, high genetic barrier to resistance and excellent safety profile [19]. Therefore, it is very crucial to organize the existing fractions of facts to create tangible evidence on comparative safety and efficacy of TDF and ZDV based cART by pooling findings of original studies with systematic review. Thus, this review is aimed to analyize and synthesize data from large observational studies for robust comparisons of efficacy, and safety of TDF based regimens with ZDV counterparts to complement evidences derived from review of randomized clinical trials.

## 2 METHODS

### 2.1 Search Strategy and Selection of Articles

The objective of this review was to systematically identify, appraise and synthesize the best available evidences on efficacy and safety of TDF based regimens as compared to ZDV regimens from observational studies.

A pre-search of review databases was conducted in 2017 to determine whether other reviews existed or protocols were under development. The Joanna Briggs Institute Database of Systematic Reviews and Implementation Reports, the Campbell Collaboration library, the National Health Centre Reviews and Dissemination databases, Health Technology Assessment, Evidence of Policy and Practice Information (EPPI-Centre) were searched using keyword and index search terms: HIV, tenofovir, and zidovudine with their MeSH terms. This search strategy described earlier, established that no other systematic reviews of observational studies was conducted on efficacy and safety of TDF based regimens as compared to ZDV based regimens.

A three-step search strategy was used to locate published and unpublished studies. First, an initial limited search of google was undertaken followed by analysis of text words contained in the title and abstract and of the index terms used to describe the articles. A second extensive search was undertaken using all the identified keywords and MeSH terms across all included databases (**Appendix I**). We searched the PubMed, EMBASE, Google Scholar, Medline, and CINHAL. We did the initial search for articles on July 11-18, 2016, and updated the results on May 13, 2017. Third, the reference lists of all identified articles were searched for additional studies that may have been missed in the electronic search. Studies identified from reference lists searches were assessed for relevance based on the study title. All authors searched each databases on the same day to be consistent. Abstracts and full reports were retrieved for studies that met the inclusion criteria.

### 2.2 Inclusion Criteria and Study Selection

The predetermined inclusion criteria were:

1. Studies published in English and conducted till May 13, 2017.
2. Observational studies
3. Data presented for comparison of TDF/FTC or 3TC with EFV or NVP and ZDV/FTC or 3TC with EFV or NVP among treatment naive adults infected with HIV-1 (age >=14 years). Lamivudine and emtricitabine (FTC) are considered as comparable in efficacy and safety for this review which is reported from previous studies [20-22], despite some recent literatures reported FTC has some advantages over 3TC [23-25].
4. There were no restrictions on country of focus.

Study selection was conducted in two stages by all authors independently; first the titles and abstracts of all potential articles were reviewed. Then, articles that passed the preliminary assessment were fully retrieved for detailed critical appraisal by two independent reviewers. In the case of disagreements during appraisal, decision was made through discussion by reviewing articles together.

Besides above mentioned inclusion criteria papers that met the inclusion criteria were critically appraised by two independent reviewers for a single study for methodological validity using standardized critical appraisal instruments from the Joanna Briggs institute meta-analysis of statistical assessment and review instrument (JBI-MAStARI) (**Appendix II**).

### 2.3 Data Extraction and Primary Study Outcomes

We extracted data from original articles if it reported at least one of the following outcomes: virologic failure, death, adverse drug events and occurrence of opportunistic infections. Mortality, occurrence of OI and virologic failure (> 1000 HIV RNA copies/ml) were considered as primary outcomes while secondary outcome was adverse drug events. We extracted outcome using the similar data extraction tool of JBI-MAStARI (**Appendix III**). All results were taken out by two independent reviewers to avoid extraction error. Data about ADEs was extracted as prevalence of adverse effects using WHO definition of ADEs or AIDS clinical trial group classification of drug toxicity or as per the report of author using set up specific criteria for assessment of ADEs. While opportunistic infection was extracted as prevalence according to WHO definition of OIs.

### 2.4 Data Analysis

Quantitative data were pooled in statistical meta-analysis using RevMan version 5.3 software. We did a fixed-effect meta-analysis to pool the Odds Ratio (OR) of the outcomes of mortality, occurrence of OI, virologic failure and ADEs. Forest plot containing OR, 95% Confidence Intervals (CI), P value, effect size, and, heterogeneity (I^2^) were constructed. P values less than 0·05 were considered statistically significant. Findings of observational studies which cannot be pooled with meta-analysis were also summarized.

## 3 RESULT

**Fig. (1):**
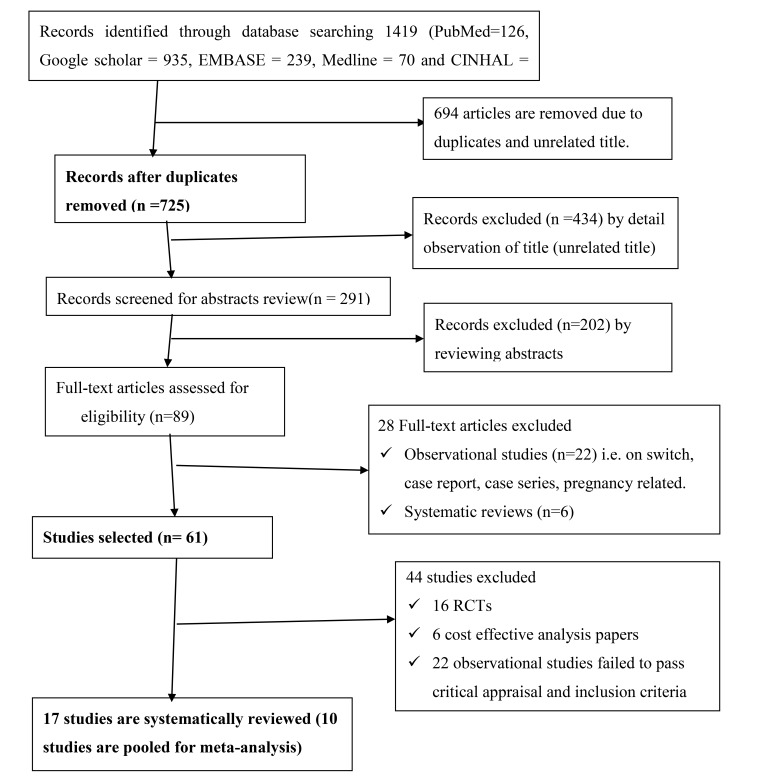
Flow chart of study selection process.

A total of 1419 articles were identified through databases searching. Of these, 694 articles were excluded as duplicates and by simple observation of titles (Fig. **1**).

### 3.1 Mortality

**Fig. (2):**
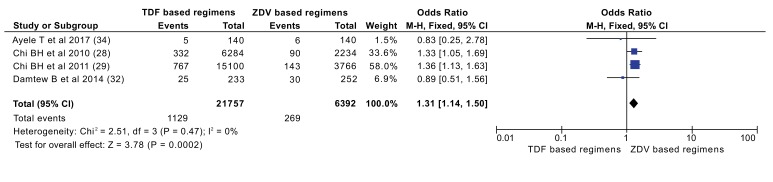
Forest Plot of Mortality effect of TDF based regimens as compared to ZDV based regimens among ART naïve HIV-1 infected patients.

Data of 21,757 patients from TDF/XTC/EFV or NVP arms and 6,392 patients from ZDV/3TC/EFV or NVP arms was assessed to compare for mortality outcome. A total of 1,129 patients (5.2%) on the TDF arms and 269 patients (4.2%) on the ZDV arms were died (Fig. **2**). Patients on TDF based regimens were 1.31 times more likely to die compared to patients on ZDV based regimens (OR: 1.31[1.14, 1.50]), (P=0.0002).

### 3.2 Virologic Failure (VF)

**Fig. (3):**
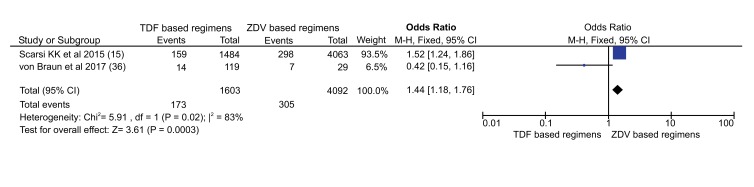
Forest Plot of VF (Viral RNA > copies/ml) in TDF based regimens as compared to ZDV based regimens among ART naïve HIV-1 infected patients.

To compare their effect on viral suppression, data of 1,603 patients treated with TDF based regimens and 4,092 patients with ZDV based regimens from two articles were included. A total of 173 patients (10.8%) on TDF arms experienced VF (Serum Viral RNA >1000 copies/ml) after 6 months of therapy on the regimens (Fig. **3**). While, 305 patients (7.5%) who were on ZDV based regimens encountered VF. Patients on TDF based regimens were 1.44 times more likely to experience VF compared to patients on ZDV based regimens (OR: 1.44 [1.18, 1.76]), (P=0.0003).

### 3.3 Adverse Drug Events

**Fig. (4):**
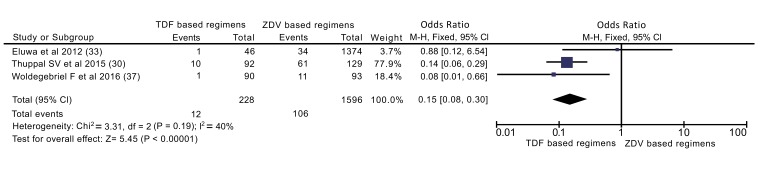
Forest Plot of ADEs occurrence in TDF based regimens as compared to ZDV based regimens among ART naïve HIV-1infected patients.

Data of 228 patients on TDF based regimens and 1,596 patients on ZDV based regimens extracted from three articles was reviewed for comparison of ADEs. A total of 12 patients (5.3%) on TDF arms and 106 patients (6.6%) on ZDV arms experienced at least one ADE (Fig. **4**). Occurrence of ADEs was significant in the ZDV based regimens as compared to TDF based regimens (P<0.00001). Patients on the TDF based regimens were 85% less likely to experience ADEs compared to patients on ZDV based regimens (OR: 0.15[0.08, 0.30]).

**Fig. (5):**
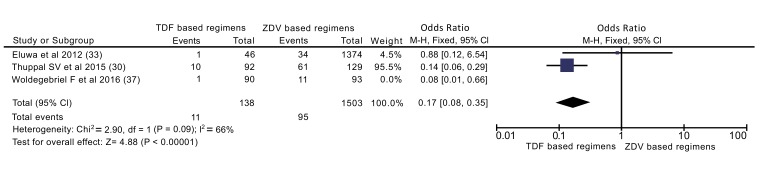
Forest Plot of ADEs occurrence in TDF based regimens as compared to ZDV based regimens among ART naïve HIV-1 infected patients in subgroup analysis excluding cross sectional study.

In addition to overall analysis, subgroup analysis was also done excluding cross sectional study (Fig. **5**). Accordingly, patients on TDF based regimens were 83% less likely to experience ADEs than ZDV based regimens (OR: 0.17[0.08, 0.35]).

### 3.4 Opportunistic Infections

**Fig. (6):**
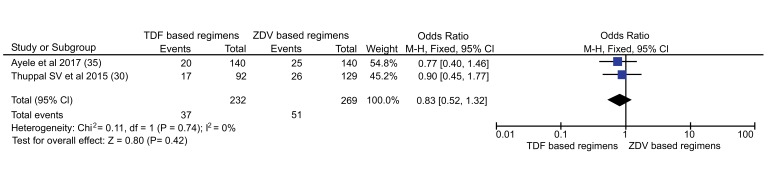
Forest Plot of OI occurrence in TDF based regimens as compared to ZDV based regimens among ART naïve HIV-1infected patients.

Data of 232 patients on TDF based regimens and 269 patients on ZDV based regimens was pooled from two articles to assess occurrence of OIs. A total of 37 patients (15.9%) on TDF arms and 51 patients (19.0%) on ZDV arms developed at least one OI (Fig. **6**). Incident of OI between the two arms was not statistically significant (p=0.42).

## 4 DISCUSSION

Getting drug combinations that is superior in its efficacy and safety is important to maintain adherence of PLWHA with cART, better quality and longevity of their life. To answer these questions, we compared TDF based with ZDV based regimens on mortality, ADEs, VF and occurrence of OIs.

To compare these regimens on mortality outcome, four observational studies were included, enrolled a total of 28,149 participants (21,757 on TDF based and 6392 on ZDV based regimens). Zidovudine based regimens had better outcome on prevention of mortality; mortality on TDF based regimens is 1.31 times higher (OR=1.31) (Fig. **4**). This finding is in contrast with the review conducted by Dadi TL *et al.*, which shows no significance between the two regimens. This difference might be due to difference in included size of participants and type of studies include in the review. The previous review included only randomized controlled trials, thus, relatively small number of participants (1858 participants) were included [18]. A review by Omeje *et al.* which included only a single study with participants of 487 also reported that no statistically significant difference in the risk of death between the two groups. Still the difference for this discrepancy might be attributed to limited number of participants derived from a single study which was unable to detect the difference between regimens [16]. Rare events like death should be identified from large participants of observational studies than clinical trials.

Only three studies, enrolled a total of 1824 participants (228 on TDF based and 1596 on ZDV based regimens) were included to compare ADEs. The result of meta-analysis shows TDF based regimens were better tolerated than ZDV counterparts. Patients who took TDF based regimens were 85% more likely to be protected than their counterparts (OR=0.15) (Fig. **6**). This difference maintained even when only cohort studies were included in analysis (OR=0.17). Similarly, finding from Omeje *et al.* reported that statistically more significant adverse events were recorded in the ZDV based regimens than TDF based regimens (9% *vs.* 4%, P = 0.02) [16]. In addition finding from Dadi TL *et al.* revealed, TDF based regimens were more tolerable than ZDV based regimen (RR = 1.06) [18]. Those findings implies that TDF based regimens are better tolerated than ZDV based regimens.

Only two studies were pooled for comparison of virologic failure ((serum RNA>1000 copies/ml)), enrolled 5695 participants (1603 on TDF based and 4093 on ZDV based regimens). Accordingly, ZDV based regimens had better outcome (OR = 1.44) (Figure 5) despite studies were heterogeneous (I^2^=83%). The heterogeneity might be partly explained by the difference in participants enrolled in each study (Table **1**).

This finding is against with previous review conducted by Omeje *et al.* where more participants on TDF group maintained plasma HIV RNA of <400 copies/ml compared to ZDV based group (84% in the TDF based group and 73% in the ZDV based group; RR 1.16; 95%CI 1.06 to 1.27) [16]. This might be due the difference in viral RNA cut-off points employed (1000 *vs.* 400 copies/ml) and the confounding effect of NNRTIs where Scarsi *et al.* used NVP while Von Braun *et al.* combined with EFV since participants were TB co-infected. However, Spaulding *et al.* reported that there were no difference between TDF and ZDV containing regimens in terms of virologic response <400 copies/ml (RR=2.04, 95% CI [0.17,24.84]) [4].

Results of two articles were analysed for comparison of occurrence of opportunistic infections. The difference between both regimens is not significant in OI outcome (OR = 0.83, 95% CI [0.52, 1.32]) (Fig. **6**). In TDF group, 16% of participants reported OI while 19% in ZDV developed OI. This proportion showed comparable OI outcome between both groups.

### 4.1 Limitations of The Study

The major limitations of this review is lack of head to head comparison of TDF and ZDV based regimens. There may be also reporting or information bias, since most of articles included in the review were conducted on secondary data. Although we searched for unpublished papers, all studies included are published papers. Thus, there is the possibility of publication bias. Because of the variability of observational study design and different methods of reporting results, there was a difficulty of pooling results. This limit the number of studies and sample size included in some outcomes. In addition variation in length of follow up among studies might affect efficacy and safety profile of each regimens.

## CONCLUSION

Pooled data showed superiority of ZDV based regimens in prevention of death and suppression of viral load. However, TDF based regimens were associated with better safety profile. But, no significant difference was observed in OI outcome between groups.

## Figures and Tables

**Figure:**
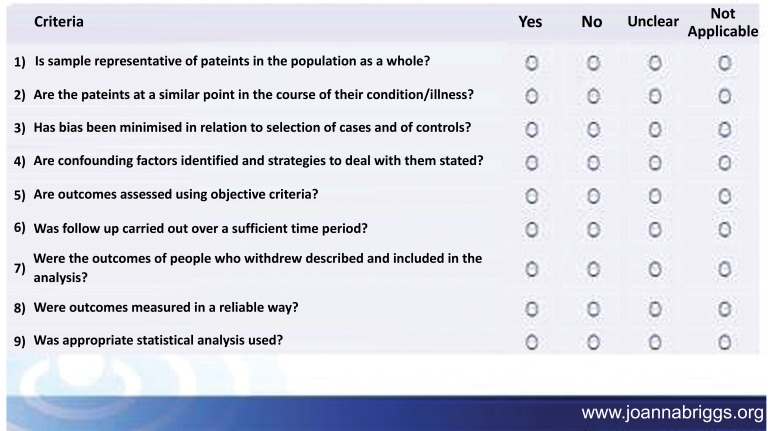


**Figure:**
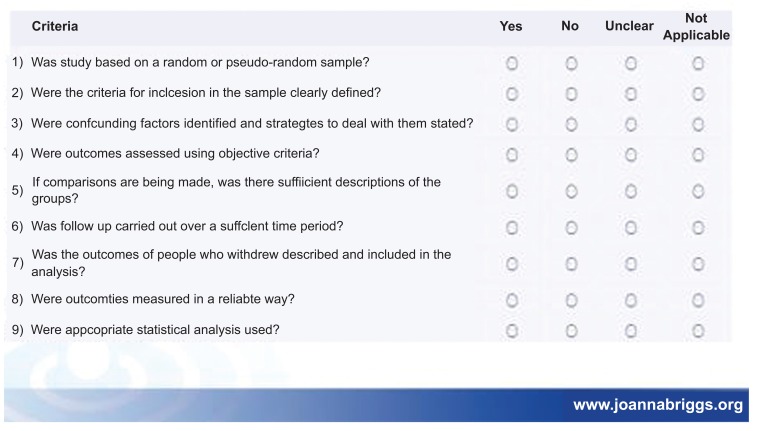


**Figure:**
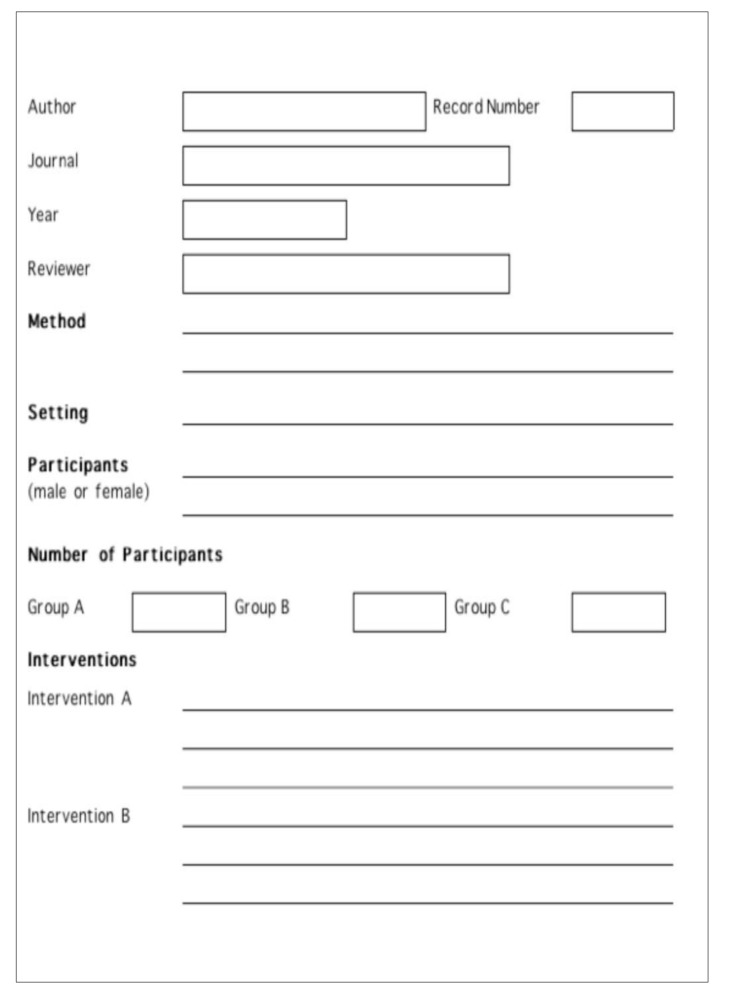


**Figure:**
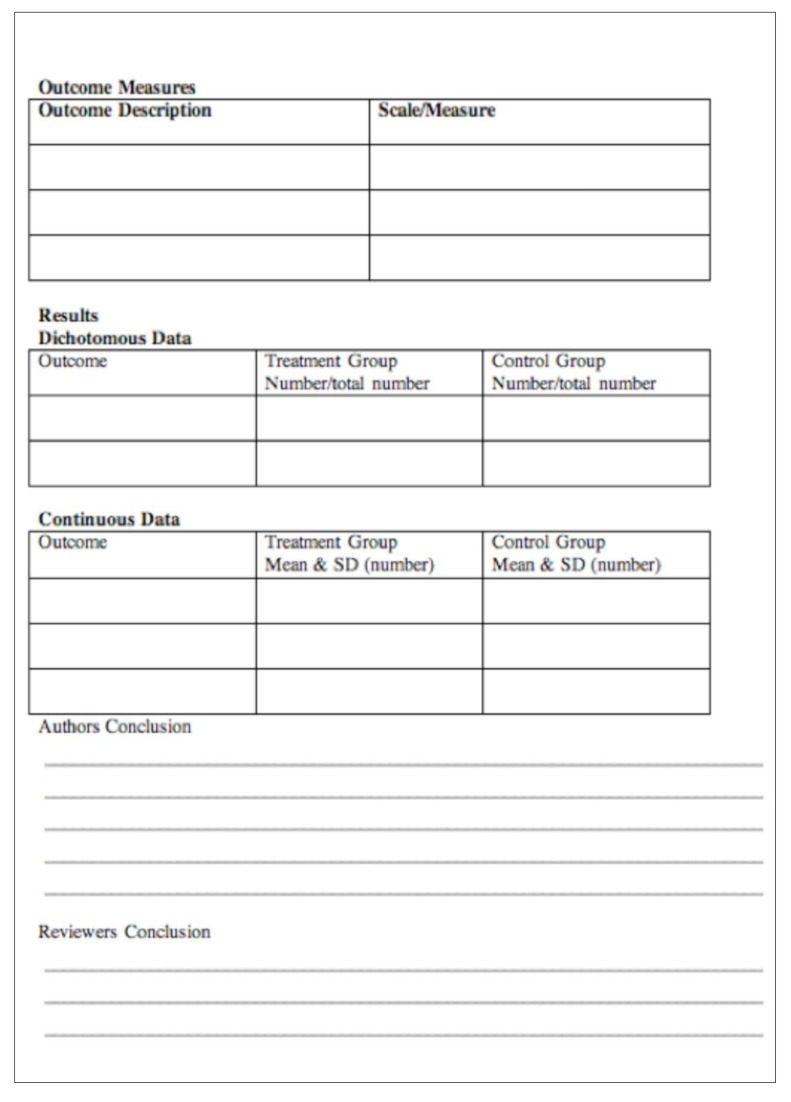


**Figure:**
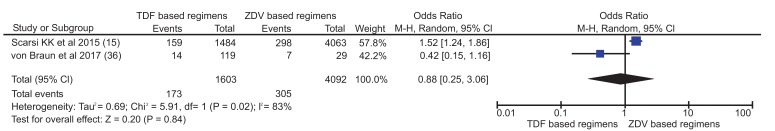


**Table 1:** characteristics of the study included in systematic review.

**Author**	**Study Design**	**Study Setting**	**Duration of Follow up**	**Data Source**	**Outcome Measure**	**Participants (type and No)**	**Findings (TDF *vs.* ZDV)**
Scarsi KK 2015 [15]	Retrospective cohort	Nigeria	105 months	Defined cohort	Virologic failure (VF) (>1000 copies/ml)	Age ≥18yr (5547)	159/1484 *vs.* 298/4063^a^ at 6 month
					ART switch not due to VF		256/1484 *vs.* 622/4063^b^
					Discontinuation*		308/1484 *vs.* 649/4063^a^
Labhartd 2015 [26]	Cross-sectional	Multicentre		Data taken from defined cohort	Virologic success (<80 copies/ml)	Age ≥16yr (1539)	930/997 *vs.* 473/542^a^
					Clinical failure		2.8% *vs.* 2.7%^b^
					Immunologic failure		4.6% *vs.* 4.8%^b^
Velen 2013 [27]	Prospective cohort	South Africa	37 months	ART program	Single drug substitution	Age ≥17yrs (2017)	10/665 *vs.* 95/1352^a^
					Mortality in PYs		9.2/100PYs *vs.* 11.1/100PYs^a^
					Loss from care in PYs		9.8/100PYs *vs.* 9.5/100PYs
					Viral suppression (<400 copies/ml at 24 months)		46% in TDF group *vs.* 42% of participants in ZDV group ^a^
Chi 2010 [28]	Retrospective cohort	Zambia	18 months	ART program	Mortality	Age >16yrs (8518)	332/6284 *vs.* 90/2234^b^
					Drug substitution in PYs		9.0/100PYs *vs.* 27.0/100PYs^a^
					Creatinine clearance (Clcr<50ml/min)		73//2759 *vs.* 5/523^a^ at 6 month while 30/960 *vs.* 7/294^b^ at 12 month respectively
					Program failure**		32.2/100PYs *vs.* 28.1/100PYs^b^
					Mean change in Clcr (ml/min)		-14.7 *vs.* -12.7^b^ at 6 month and -22.0 *vs.* -23.7^b^ at 12 month
Chi 2011 [29]	Retrospective cohort	Zambia	40 months	ART program	Mortality	Age >16yrs (18866)	767/15100 *vs.* 143/3766^b^
					Program failure**		4359/15100 *vs.* 1412/3766^b^
Thuppal 2015 [30]	Retrospective cohort	India	36 months	ART program	Adverse drug events	Adults (221)	10/92 *vs.* 61/129^a^
					Opportunistic infections		17/92 *vs.* 26/129^b^
					Hospitalization		18/92 *vs.* 30/129^b^
					Mean change in CD4 (SD)		388(198) *vs.* 359(220)^b^
					Mean change in BMI (SD)		3.6(3) *vs.* 1.8 (2.5)^a^
Amoroso 2012 [31]	cross-sectional	Multicentre	48 months	Defined cohort	Viral suppression (<400 copies/ml) after 9 months	Age ≥16yrs (1819)	597/668 *vs.* 1008/1151^a^
Damtew 2014 [32]	Retrospective cohort	Ethiopia	60 months	ART program	Mortality	Age ≥15yr (485)	25/233 *vs.* 30/252^b^
Eluwa 2012 [33]	Retrospective cohort	Nigeria	36 months	ART program	Adverse drug events	Age ≥15yr (1420)	1/46 *vs.* 34/1374^a^
Ayele T 2017 [34]	Retrospective cohort	Ethiopia	24 months	ART program	Mortality	Age ≥14yr (280)	5/140 *vs.* 6/140^b^
					Opportunistic infections		20/140 *vs.* 25/140^a^
Ayele 2017 [35]	Retrospective cohort	Ethiopia	24 months	ART program	Mean change in CD4 (SD)	Age ≥14yr (280)	321.7(164.8) *vs.* 299.4(126.1)^a^
von Braun 2017 [36]	Cross-sectional	Uganda	24 months	Defined cohort	VF (>1000 copies/ml)	TB/HIV co-infected adults (Age ≥18yrs) (148)	14/119 *vs.* 7/29^c^ at 6 month
Woldegebriel 2016 [37]	Cross-sectional	Ethiopia	96 months	ART program	Adverse drug events	Age ≥18yrs (183)	1/90 *vs.* 11/93^a^
Parkes-ratanshi 2015 [38]	Nested cohort	Uganda	48 months	Defined cohort	Anemia (Hgb<6.5g/dl)	Adults (224)	5/63 *vs.* 18/161^c^
					Mean change in Hgb (IQR)		0.84(0.51-1.45) *vs.* 1(0.91-1.52)^c^ at 48 weeks
PrayGod 2017 [39]	Cross-sectional	Tanzania	40 months	Defined cohort	prediabetes or diabetes development	Malnourished adults (260)	30/135 *vs.* 29/125^b^
Baynes 2017 [40]	Cross-sectional []	Ethiopia	60 months	ART program	Serum creatinine (SD)	Age>15years (245)	0.83(0.36) *vs.* 0.87(0.38)^a^
					Blood urea nitrogen		11.74(4.17) *vs.* 14.86(7.53)^a^
					ALT		31.1(4.2) *vs.* 32.2 (2.3)^b^
Biset 2016 [41]	Cross-sectional	Ethiopia	42 months	ART program	Treatment failure	Age ≥18yr (330)	11/155 *vs.* 3/175^c^
					Immunologic failure		10/155 *vs.* 3/175^c^
					VF (<5000 copies/ml)		10/155 *vs.* 2/175^c^

*Death, lost follow up, transferred, withdrawal, **death, lost follow up, withdrawal ALT, alanine aminotransferase; BMI, body mass index; IQR, inter quartile range; Hgb, haemoglobin; PYs: person years; SD, standard deviation; VF, Virologic failure, ^a^ statistically significant (P<0.05), ^b^ not significant, ^c^ not reported

## LIST OF ABBREVIATIONS

ADE: = Adverse Drug Event
AIDS: = Acquired Immunodeficiency Syndrome
ART: = Antiretroviral Therapy
cART: = Combination of Antiretroviral Therapy
CI: = Confidence Intervals
EFV: = Efavirenz
HIV: = Human Immunodefiency Virus
NVP: = Nevirapine
OI: = Opportunistic Infections
PLWHA: = People Living with HIV/AIDS
RR: = Relative Risk
TDF: = Tenofovir Disoproxil Fumarate
TDF/3TC (FTC)/EFV: = Tenofovir Disoproxil Fumarate plus Lamivudine or Emtricitabine plus Efavirenz
TDF/3TC (FTC)/NVP: = Tenofovir Disoproxil Fumarate plus Lamivudine or Emtricitabine plus Nevirapine
VF: = Virologic failure
WHO: = World Health Organization
ZDV: = Zidovudine
ZDV/3TC/NVP: = Zidovudine plus Lamivudine plus nevirapine
ZDV/3TC/EFV: = Zidovudine plus Lamivudine plus Efavirenz

## APPENDIX I: SEARCH STRATEGY FOR PUBMED

((((((((tenofovir) OR (R) -9- (2-phosphonylmethoxypropyl) adenine) OR 9- (2-Phosphonomethoxypropyl) adenine) OR 9- (2-Phosphonylmethoxypropyl) adenine) OR 9- (2-Phosphonylmethoxypropyl) adenine, isomer) OR 9- (2-Phosphonylmethoxypropyl) adenine, (R) isomer t357098) OR 9- (2-Phosphonylmethoxypropyl) adenine, (S) isomer) OR 9-PMPA (tenofovir)) OR tenofovir disoproxil) OR tenofovir disoproxil fumarate) OR viread)) AND (zidovudine) OR 3'-Azido-2',3'-Dideoxythymidine) OR 3'-Azido-3'-deoxythymidine) OR Azidothymidine) OR AZT (Antiviral)) OR AZT Antiviral) OR BW A509U) OR BWA-509U) OR Retrovir)) AND ((((((((((((((((((HIV) OR Acquired Immune Deficiency Syndrome Virus) OR Acquired Immunodeficiency Syndrome Virus) OR AIDS Virus) OR HTLV-III) OR Human Immunodeficiency Virus) OR Human Immunodeficiency Viruses) OR Human T Cell Lymphotropic Virus Type III) OR Human T Lymphotropic Virus Type III) OR Human T-Cell Leukemia Virus Type III) OR Human T-Cell Lymphotropic Virus Type III) OR Human T-Lymphotropic Virus Type III) OR Immunodeficiency Virus, Human) OR Immunodeficiency Viruses, Human) OR LAV-HTLV-III) OR Lymphadenopathy-Associated Virus) OR Virus, Human Immunodeficiency) OR Viruses, Human Immunodeficiency) Filters activated: Clinical Trial, Review, Systematic Reviews, Observational Study, Comparative Study, Meta-Analysis, Humans, English, Adult: 19+ years, Adolescent: 13-18 years.

## APPENDIX II: APPRAISAL INSTRUMENT

JBI Critical Appraisal Checklist for Cohort/Case Control Studies

JBI Critical Appraisal Checklist For Cross-Sectional Studies
